# Di-μ-hydroxido-κ^4^ *O*:*O*-μ-trifluoro­methane­sulfonato-κ^2^ *O*:*O*′-bis­[(5,5′-dimethyl-2,2-bipyridine-κ^2^ *N*,*N*′)(η^5^-penta­methyl­cyclo­penta­dien­yl)ytterbium(III)] tetra­phenyl­borate 5,5′-dimethyl-2,2-bipyridine

**DOI:** 10.1107/S1600536808002791

**Published:** 2008-01-30

**Authors:** Daniel Kazhdan

**Affiliations:** a Chemistry Department and Chemical Sciences Division of Lawrence Berkeley National Laboratory, University of California, Berkeley, CA 94720, USA

## Abstract

The title compound, [Yb_2_(CF_3_O_3_S)(C_10_H_15_)_2_(OH)_2_(C_12_H_12_N_2_)_2_](C_24_H_20_B)·C_12_H_12_N_2_, crystallizes as a half-sandwich complex with a bridging trifluoro­methane­sulfonate as well as two bridging hydroxide groups. The bound bipyridine ligands have N—C—C—N torsion angles of 13.1 (9) and −12.1 (8)°. The structure also contains an uncoordinated 5,5′-dimethyl-2,2′-bipyridine molecule with an N—C—C—N torsion angle of 169.5 (7)°. The triply bridged Yb centers are 3.5990 (4) Å apart. The Yb—N bond lengths are in the range 2.389 (6)–2.424 (5) Å.

## Related literature

For related literature, see: Allen (2002 ▶); van Albada *et al.* (2005 ▶); Schultz *et al.* (2002 ▶).
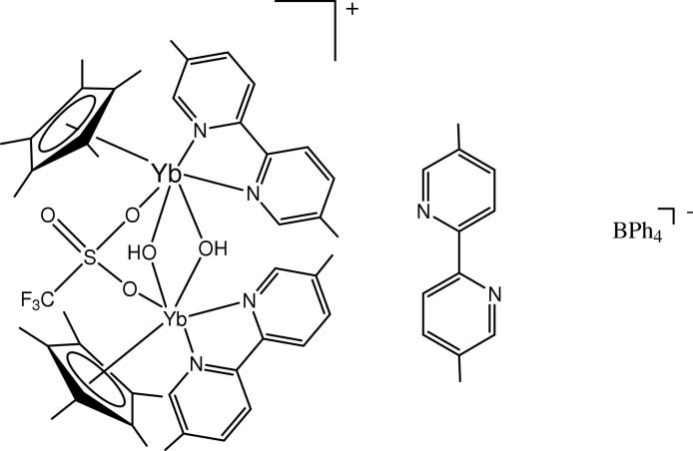


## Experimental

### 

#### Crystal data

[Yb_2_(CF_3_O_3_S)(C_10_H_15_)_2_(OH)_2_(C_12_H_12_N_2_)_2_](C_24_H_20_B)·C_12_H_12_N_2_
*M* *_r_* = 1671.57
Triclinic, 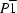

*a* = 13.0458 (8) Å
*b* = 13.7179 (9) Å
*c* = 23.3306 (15) Å
α = 87.215 (1)°
β = 86.669 (1)°
γ = 68.161 (1)°
*V* = 3867.4 (4) Å^3^
*Z* = 2
Mo *K*α radiation
μ = 2.49 mm^−1^
*T* = 130 (1) K
0.15 × 0.13 × 0.08 mm

#### Data collection

Bruker APEX diffractometer
Absorption correction: multi-scan (using intensity measurements; Blessing, 1995 ▶) *T*_min_ = 0.691, *T*_max_ = 0.819
20924 measured reflections
15784 independent reflections
9459 reflections with *F* ^2^ > 3σ(*F* ^2^)
*R*_int_ = 0.021

#### Refinement

*R*[*F* ^2^ > 2σ(*F* ^2^)] = 0.040
*wR*(*F* ^2^) = 0.040
*S* = 1.39
9459 reflections
892 parameters
H-atom parameters constrained
Δρ_max_ = 1.59 e Å^−3^
Δρ_min_ = −2.03 e Å^−3^

### 

Data collection: *SMART* (Bruker, 2002 ▶); cell refinement: *SAINT* (Bruker, 2002 ▶); data reduction: *SAINT*; program(s) used to solve structure: *SIR97* (Altomare *et al.*, 1999 ▶); program(s) used to refine structure: *TEXSAN* (MSC/Rigaku, 1998 ▶); molecular graphics: *TEXSAN*; software used to prepare material for publication: *TEXSAN*.

## Supplementary Material

Crystal structure: contains datablocks global, I. DOI: 10.1107/S1600536808002791/pv2065sup1.cif

Structure factors: contains datablocks I. DOI: 10.1107/S1600536808002791/pv2065Isup2.hkl

Additional supplementary materials:  crystallographic information; 3D view; checkCIF report

## Figures and Tables

**Table:** *Cg*1 and *Cg*2 are the centroids of the C1–C5 and C6–C10 rings, respectively.

Yb1—Yb2	3.5990 (4)
Yb1—O1	2.255 (4)
Yb1—O2	2.207 (4)
Yb1—O3	2.361 (4)
Yb1—N1	2.408 (5)
Yb1—N2	2.424 (5)
Yb1—*Cg*1	2.3750 (3)
Yb2—O1	2.295 (5)
Yb2—O2	2.214 (4)
Yb2—O4	2.332 (5)
Yb2—N3	2.408 (6)
Yb2—N4	2.389 (6)
Yb2—*Cg*2	2.3585 (3)

**Table:** 

Yb1—O1—Yb2	104.5 (2)
Yb1—O2—Yb2	109.0 (2)

**Fig. 1.**
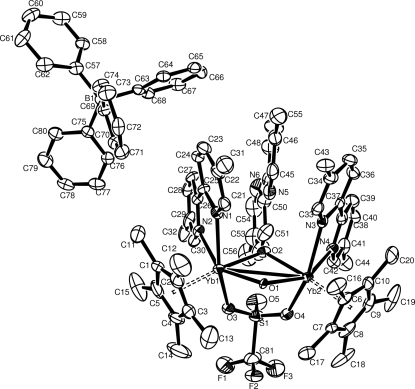
A view of the title compound with displacement ellipsoids drawn at the 50% probability level; hydrogen atoms have been omitted for clarity.

## supplementary crystallographic information

### Comment

The title compound, (I), is presented in Fig. 1, wherein, the cyclopentadienyl ligands represented by *Cg*1 and *Cg*2, are the centroids of the rings defined by C1—C5 and C6—C10, respectively. The two pyridine rings of the bipyridine (bpy) ligands are not coplanar; the torsion angles N—C—C—N are 12–13°. The Yb(III)-N distances of 2.389 (6)–2.424 (5) Å in (I) are not uncommon for neutral bpy bound to a Ytterbium center (Schultz *et al.*, 2002). The trifluoromethanesulfonate anion is bridging. The uncoordinated 5,5'-dimethyl-2,2'-bipyridine is the second structure (van Albada *et al.*, 2005) of uncoordinated 5,5'-dimethyl-2,2'-bipyridine in the Cambridge Structural Database (Allen, 2002).

### Experimental

Impure NaBPh_4_ (1.00 g, 3.13 mmol) purchased from Aldrich was added to ([Cp*_2_Yb(5,5'-dimethyl-2,2'-bipyridine)][OTf] (1.16 g, 1.49 mmol) in the presence of 5,5'-dimethyl-2,2'-bipyridine (0.50 g, 2.71 mmol) in ether. The solution was stirred overnight and the ether was filtered off. The yellow residue was dissolved in dichloromethane, filtered, and then layered with pentane. After 3 days dark yellow crystals were obtained. Yield 0.86 g (77%).

### Refinement

All non-hydrogen atoms were refined anisotropically. Hydrogen atoms bonded to C-atoms were included in the refinements at geometrically idealized positions with C—H = 0.95 Å and *U*_iso_ = 1.2 times *U*_eq_ of the parent atoms. The hydrxyl H-atoms were located from a difference map and included in the refinements at those positions with *U*_iso_ = 1.2 times *U*_eq_ of the O-atoms. The final difference map showed residual electron denities in the close proximity of Yb atoms.

### Crystal data

**Table:**

[Yb_2_(CF_3_O_3_S)(C_10_H_15_)_2_(OH)_2_(C_12_H_12_N_2_)_2_](C_24_H_20_B)·C_12_H_12_N_2_	*Z* = 2
*M**_r_* = 1671.57	*F*_000_ = 1688.00
Triclinic, *P*1	*D*_x_ = 1.435 Mg m^−^^3^
Hall symbol: -P 1	Melting point: 518 (with decomposition) K
*a* = 13.0458 (8) Å	Mo *K*α radiation λ = 0.7107 Å
*b* = 13.7179 (9) Å	Cell parameters from 4695 reflections
*c* = 23.3306 (15) Å	θ = 2.4–24.8º
α = 87.215 (1)º	µ = 2.49 mm^−^^1^
β = 86.669 (1)º	*T* = 130 (1) K
γ = 68.161 (1)º	Block, yellow
*V* = 3867.4 (4) Å^3^	0.15 × 0.13 × 0.08 mm

### Data collection

**Table:**

Bruker APEX diffractometer	9459 reflections with *F*^2^ > 3σ(*F*^2^)
ω scans	*R*_int_ = 0.021
Absorption correction: multi-scan(Blessing, 1995)	θ_max_ = 26.4º
*T*_min_ = 0.691, *T*_max_ = 0.819	*h* = 0→16
20924 measured reflections	*k* = −15→17
15784 independent reflections	*l* = −28→29

### Refinement

**Table:**

Refinement on *F*	H-atom parameters constrained
*R*[*F*^2^ > 2σ(*F*^2^)] = 0.040	*w* = 1/[σ^2^(*F*_o_) + 0.00022|*F*_o_|^2^]
*wR*(*F*^2^) = 0.040	(Δ/σ)_max_ = 0.002
*S* = 1.39	Δρ_max_ = 1.59 e Å^−^^3^
9459 reflections	Δρ_min_ = −2.03 e Å^−^^3^
892 parameters	Extinction correction: none

### Special details

**Table:**

Refinement. Refinement using reflections with *F*^2^ > 3.0 σ(*F*^2^). The weighted *R*-factor (*wR*), goodness of fit (*S*) and *R*-factor (gt) are based on *F*, with *F* set to zero for negative *F*. The threshold expression of *F*^2^ > 3.0 σ(*F*^2^) is used only for calculating *R*-factor (gt).

### Fractional atomic coordinates and isotropic or equivalent isotropic displacement parameters (Å^2^)

**Table:**

	*x*	*y*	*z*	*U*_iso_*/*U*_eq_	
Yb1	0.70164 (2)	0.49056 (2)	0.66984 (1)	0.0250 (1)	
Yb2	0.80600 (2)	0.69462 (2)	0.69016 (1)	0.01960 (9)	
S1	0.6741 (1)	0.6896 (2)	0.56502 (7)	0.0321 (5)	
F1	0.6795 (4)	0.6389 (4)	0.4581 (2)	0.069 (2)	
F2	0.8393 (4)	0.6074 (4)	0.4912 (2)	0.057 (2)	
F3	0.7333 (4)	0.7659 (4)	0.4700 (2)	0.070 (2)	
O1	0.8570 (3)	0.5154 (4)	0.6904 (2)	0.026 (1)	
O2	0.6559 (3)	0.6550 (3)	0.6948 (2)	0.023 (1)	
O3	0.6775 (4)	0.5851 (4)	0.5810 (2)	0.033 (1)	
O4	0.7518 (4)	0.7203 (4)	0.5953 (2)	0.031 (1)	
O5	0.5672 (4)	0.7675 (4)	0.5602 (2)	0.055 (2)	
N1	0.6535 (4)	0.4642 (4)	0.7690 (2)	0.024 (2)	
N2	0.5035 (4)	0.5468 (4)	0.6893 (2)	0.027 (2)	
N3	0.7635 (4)	0.7154 (4)	0.7917 (2)	0.022 (2)	
N4	0.6725 (4)	0.8647 (4)	0.7116 (2)	0.025 (2)	
N5	0.4492 (5)	0.7822 (5)	0.7625 (3)	0.039 (2)	
N6	0.1947 (5)	0.9558 (6)	0.7027 (3)	0.058 (3)	
C1	0.7007 (6)	0.2975 (6)	0.6703 (3)	0.032 (2)	
C2	0.8129 (6)	0.2807 (6)	0.6790 (4)	0.039 (2)	
C3	0.8534 (7)	0.3152 (6)	0.6291 (4)	0.048 (3)	
C4	0.7716 (8)	0.3504 (6)	0.5889 (3)	0.048 (3)	
C5	0.6736 (7)	0.3398 (7)	0.6156 (4)	0.049 (3)	
C6	1.0124 (5)	0.6507 (5)	0.7130 (3)	0.026 (2)	
C7	1.0094 (5)	0.6468 (5)	0.6520 (3)	0.025 (2)	
C8	0.9562 (5)	0.7504 (6)	0.6314 (3)	0.036 (2)	
C9	0.9256 (5)	0.8182 (6)	0.6779 (4)	0.038 (2)	
C10	0.9611 (5)	0.7561 (6)	0.7283 (3)	0.033 (2)	
C11	0.6294 (7)	0.2602 (7)	0.7125 (4)	0.055 (3)	
C12	0.8793 (7)	0.2231 (7)	0.7285 (4)	0.068 (3)	
C13	0.9738 (8)	0.3048 (8)	0.6175 (6)	0.096 (4)	
C14	0.7870 (10)	0.3800 (9)	0.5268 (4)	0.114 (5)	
C15	0.5671 (10)	0.3599 (8)	0.5869 (5)	0.095 (5)	
C16	1.0650 (5)	0.5600 (7)	0.7532 (3)	0.045 (3)	
C17	1.0620 (6)	0.5487 (7)	0.6176 (4)	0.051 (3)	
C18	0.9452 (7)	0.7837 (9)	0.5681 (4)	0.073 (4)	
C19	0.8794 (7)	0.9355 (7)	0.6749 (5)	0.086 (4)	
C20	0.9583 (6)	0.8002 (8)	0.7858 (4)	0.065 (3)	
C21	0.7309 (5)	0.4158 (6)	0.8074 (3)	0.029 (2)	
C22	0.7101 (6)	0.3799 (6)	0.8621 (3)	0.033 (2)	
C23	0.5998 (6)	0.4030 (6)	0.8778 (3)	0.036 (2)	
C24	0.5188 (6)	0.4562 (6)	0.8410 (3)	0.031 (2)	
C25	0.5473 (5)	0.4847 (5)	0.7859 (3)	0.025 (2)	
C26	0.4645 (5)	0.5410 (5)	0.7435 (3)	0.025 (2)	
C27	0.3517 (6)	0.5908 (6)	0.7578 (3)	0.031 (2)	
C28	0.2812 (6)	0.6459 (6)	0.7163 (4)	0.040 (3)	
C29	0.3192 (6)	0.6520 (6)	0.6605 (3)	0.038 (2)	
C30	0.4317 (6)	0.6014 (6)	0.6494 (3)	0.033 (2)	
C31	0.8036 (7)	0.3183 (6)	0.8995 (3)	0.048 (3)	
C32	0.2455 (6)	0.7120 (7)	0.6133 (4)	0.055 (3)	
C33	0.8136 (5)	0.6400 (5)	0.8300 (3)	0.026 (2)	
C34	0.8072 (5)	0.6517 (6)	0.8883 (3)	0.031 (2)	
C35	0.7411 (6)	0.7509 (6)	0.9083 (3)	0.032 (2)	
C36	0.6838 (5)	0.8293 (6)	0.8700 (3)	0.032 (2)	
C37	0.6967 (5)	0.8113 (5)	0.8113 (3)	0.025 (2)	
C38	0.6377 (5)	0.8902 (5)	0.7662 (3)	0.027 (2)	
C39	0.5509 (6)	0.9833 (6)	0.7793 (3)	0.035 (2)	
C40	0.5011 (6)	1.0516 (6)	0.7352 (4)	0.038 (2)	
C41	0.5353 (6)	1.0275 (6)	0.6787 (3)	0.035 (2)	
C42	0.6210 (5)	0.9328 (5)	0.6695 (3)	0.028 (2)	
C43	0.8729 (6)	0.5626 (6)	0.9275 (3)	0.039 (2)	
C44	0.4817 (7)	1.0988 (6)	0.6284 (4)	0.051 (3)	
C45	0.4850 (6)	0.7391 (6)	0.8133 (4)	0.039 (3)	
C46	0.4236 (6)	0.7606 (6)	0.8655 (3)	0.041 (3)	
C47	0.3186 (8)	0.8331 (8)	0.8627 (4)	0.061 (3)	
C48	0.2765 (7)	0.8790 (7)	0.8107 (4)	0.053 (3)	
C49	0.3443 (6)	0.8532 (6)	0.7613 (4)	0.038 (2)	
C50	0.3040 (6)	0.8982 (6)	0.7049 (4)	0.043 (3)	
C51	0.3734 (7)	0.8815 (7)	0.6555 (4)	0.052 (3)	
C52	0.3297 (8)	0.9247 (8)	0.6025 (4)	0.065 (4)	
C53	0.2166 (8)	0.9837 (7)	0.5999 (4)	0.064 (3)	
C54	0.1563 (7)	0.9950 (7)	0.6507 (5)	0.064 (3)	
C55	0.4730 (7)	0.7060 (7)	0.9196 (4)	0.058 (3)	
C56	0.1637 (9)	1.0264 (8)	0.5443 (5)	0.087 (4)	
C57	0.2234 (5)	0.2202 (6)	0.9247 (3)	0.026 (2)	
C58	0.1245 (5)	0.2877 (5)	0.9514 (3)	0.028 (2)	
C59	0.0499 (6)	0.2521 (7)	0.9809 (3)	0.038 (2)	
C60	0.0699 (6)	0.1457 (7)	0.9850 (3)	0.044 (3)	
C61	0.1645 (6)	0.0774 (6)	0.9592 (3)	0.044 (3)	
C62	0.2392 (5)	0.1137 (6)	0.9298 (3)	0.032 (2)	
C63	0.2899 (5)	0.3824 (5)	0.9082 (3)	0.023 (2)	
C64	0.3516 (5)	0.4014 (5)	0.9507 (3)	0.026 (2)	
C65	0.3352 (6)	0.5008 (6)	0.9688 (3)	0.031 (2)	
C66	0.2551 (6)	0.5878 (6)	0.9450 (3)	0.033 (2)	
C67	0.1903 (6)	0.5736 (6)	0.9034 (3)	0.035 (2)	
C68	0.2085 (5)	0.4723 (6)	0.8861 (3)	0.028 (2)	
C69	0.4393 (5)	0.1896 (5)	0.8980 (3)	0.023 (2)	
C70	0.5256 (5)	0.1871 (5)	0.8590 (3)	0.028 (2)	
C71	0.6363 (5)	0.1338 (6)	0.8700 (3)	0.033 (2)	
C72	0.6659 (5)	0.0811 (5)	0.9224 (3)	0.029 (2)	
C73	0.5838 (6)	0.0808 (6)	0.9619 (3)	0.035 (2)	
C74	0.4730 (5)	0.1345 (5)	0.9499 (3)	0.028 (2)	
C75	0.2831 (5)	0.2681 (5)	0.8183 (3)	0.024 (2)	
C76	0.3178 (5)	0.3285 (5)	0.7773 (3)	0.025 (2)	
C77	0.2980 (6)	0.3321 (6)	0.7191 (3)	0.033 (2)	
C78	0.2408 (6)	0.2754 (6)	0.6998 (3)	0.035 (2)	
C79	0.2048 (6)	0.2150 (6)	0.7380 (3)	0.040 (2)	
C80	0.2258 (6)	0.2116 (6)	0.7963 (3)	0.033 (2)	
C81	0.7344 (7)	0.6750 (8)	0.4919 (3)	0.049 (3)	
B1	0.3097 (6)	0.2631 (6)	0.8869 (3)	0.023 (2)	
H1	0.9049	0.4718	0.7069	0.0370*	
H2	0.5838	0.7074	0.6928	0.0370*	
H3	0.6660	0.1874	0.7210	0.0665*	
H4	0.5607	0.2717	0.6962	0.0665*	
H5	0.6168	0.2982	0.7469	0.0665*	
H6	0.8334	0.2024	0.7556	0.0820*	
H7	0.9083	0.2676	0.7462	0.0820*	
H8	0.9383	0.1625	0.7151	0.0820*	
H9	1.0044	0.3105	0.6523	0.1148*	
H10	1.0145	0.2385	0.6014	0.1148*	
H11	0.9760	0.3592	0.5911	0.1148*	
H12	0.8402	0.3217	0.5080	0.1368*	
H13	0.8109	0.4374	0.5246	0.1368*	
H14	0.7183	0.3996	0.5086	0.1368*	
H15	0.5714	0.2983	0.5682	0.1138*	
H16	0.5076	0.3780	0.6149	0.1138*	
H17	0.5547	0.4160	0.5593	0.1138*	
H18	1.0896	0.4966	0.7327	0.0540*	
H19	1.0126	0.5577	0.7827	0.0540*	
H20	1.1262	0.5679	0.7696	0.0540*	
H21	1.0751	0.4885	0.6423	0.0609*	
H22	1.0139	0.5483	0.5886	0.0609*	
H23	1.1301	0.5475	0.6002	0.0609*	
H24	0.8768	0.8405	0.5631	0.0876*	
H25	1.0043	0.8051	0.5555	0.0876*	
H26	0.9475	0.7261	0.5463	0.0876*	
H27	0.8138	0.9609	0.6986	0.1033*	
H28	0.9321	0.9612	0.6878	0.1033*	
H29	0.8629	0.9590	0.6363	0.1033*	
H30	0.9794	0.7447	0.8139	0.0783*	
H31	0.8856	0.8478	0.7951	0.0783*	
H32	1.0082	0.8360	0.7850	0.0783*	
H33	0.8054	0.4051	0.7963	0.0342*	
H34	0.5803	0.3815	0.9148	0.0430*	
H35	0.4432	0.4737	0.8528	0.0373*	
H36	0.3243	0.5863	0.7961	0.0366*	
H37	0.2048	0.6804	0.7261	0.0478*	
H38	0.4600	0.6053	0.6112	0.0399*	
H39	0.8067	0.3607	0.9299	0.0574*	
H40	0.8712	0.2974	0.8773	0.0574*	
H41	0.7921	0.2577	0.9151	0.0574*	
H42	0.2863	0.7384	0.5860	0.0657*	
H43	0.1850	0.7689	0.6291	0.0657*	
H44	0.2184	0.6667	0.5949	0.0657*	
H45	0.8574	0.5728	0.8158	0.0312*	
H46	0.7357	0.7642	0.9481	0.0389*	
H47	0.6356	0.8956	0.8837	0.0388*	
H48	0.5263	0.9994	0.8180	0.0415*	
H49	0.4426	1.1161	0.7437	0.0453*	
H50	0.6453	0.9145	0.6310	0.0331*	
H51	0.8942	0.4982	0.9081	0.0472*	
H52	0.8289	0.5599	0.9610	0.0472*	
H53	0.9370	0.5733	0.9381	0.0472*	
H54	0.5096	1.0633	0.5935	0.0615*	
H55	0.4039	1.1168	0.6318	0.0615*	
H56	0.4979	1.1608	0.6283	0.0615*	
H57	0.5586	0.6893	0.8143	0.0470*	
H58	0.2736	0.8522	0.8969	0.0735*	
H59	0.2024	0.9275	0.8088	0.0633*	
H60	0.4503	0.8417	0.6578	0.0614*	
H61	0.3764	0.9139	0.5688	0.0775*	
H62	0.0793	1.0346	0.6493	0.0771*	
H63	0.5471	0.6603	0.9116	0.0701*	
H64	0.4314	0.6663	0.9353	0.0701*	
H65	0.4720	0.7565	0.9463	0.0701*	
H66	0.1941	1.0747	0.5274	0.1039*	
H67	0.1771	0.9703	0.5190	0.1039*	
H68	0.0863	1.0613	0.5510	0.1039*	
H69	0.1085	0.3612	0.9490	0.0338*	
H70	−0.0154	0.3008	0.9984	0.0454*	
H71	0.0189	0.1206	1.0053	0.0524*	
H72	0.1791	0.0041	0.9616	0.0522*	
H73	0.3039	0.0640	0.9122	0.0387*	
H74	0.4075	0.3430	0.9679	0.0308*	
H75	0.3794	0.5091	0.9977	0.0372*	
H76	0.2443	0.6563	0.9569	0.0392*	
H77	0.1340	0.6325	0.8868	0.0421*	
H78	0.1631	0.4642	0.8577	0.0332*	
H79	0.5075	0.2238	0.8231	0.0341*	
H80	0.6918	0.1332	0.8417	0.0394*	
H81	0.7415	0.0460	0.9308	0.0349*	
H82	0.6026	0.0439	0.9976	0.0421*	
H83	0.4180	0.1335	0.9782	0.0341*	
H84	0.3570	0.3692	0.7899	0.0297*	
H85	0.3240	0.3739	0.6930	0.0394*	
H86	0.2262	0.2780	0.6602	0.0420*	
H87	0.1653	0.1752	0.7249	0.0483*	
H88	0.2000	0.1689	0.8219	0.0399*	

### Atomic displacement parameters (Å^2^)

**Table:**

	*U*^11^	*U*^22^	*U*^33^	*U*^12^	*U*^13^	*U*^23^
Yb1	0.0231 (2)	0.0338 (2)	0.0214 (2)	−0.0146 (2)	0.0077 (1)	−0.0081 (2)
Yb2	0.0136 (2)	0.0213 (2)	0.0218 (2)	−0.0043 (1)	0.0025 (1)	−0.0020 (1)
S1	0.028 (1)	0.046 (1)	0.0212 (10)	−0.0128 (9)	0.0000 (8)	−0.0007 (9)
F1	0.090 (4)	0.109 (5)	0.027 (3)	−0.059 (4)	−0.009 (3)	−0.006 (3)
F2	0.060 (3)	0.079 (4)	0.038 (3)	−0.035 (3)	0.021 (2)	−0.020 (3)
F3	0.116 (5)	0.081 (4)	0.030 (3)	−0.058 (4)	−0.001 (3)	0.012 (3)
O1	0.035 (3)	0.040 (3)	0.019 (3)	−0.032 (3)	0.007 (2)	−0.009 (2)
O2	0.013 (2)	0.031 (3)	0.022 (3)	−0.005 (2)	−0.002 (2)	0.003 (2)
O3	0.039 (3)	0.047 (3)	0.021 (3)	−0.025 (3)	0.003 (2)	−0.007 (2)
O4	0.031 (3)	0.040 (3)	0.027 (3)	−0.020 (2)	0.003 (2)	−0.001 (2)
O5	0.031 (3)	0.063 (4)	0.057 (4)	−0.002 (3)	−0.008 (3)	0.009 (3)
N1	0.019 (3)	0.026 (3)	0.027 (3)	−0.007 (3)	−0.001 (2)	−0.006 (3)
N2	0.027 (3)	0.033 (4)	0.027 (4)	−0.018 (3)	0.003 (3)	−0.003 (3)
N3	0.016 (3)	0.020 (3)	0.031 (3)	−0.007 (3)	0.001 (2)	−0.003 (3)
N4	0.019 (3)	0.030 (4)	0.027 (3)	−0.011 (3)	0.003 (2)	−0.003 (3)
N5	0.033 (4)	0.036 (4)	0.052 (5)	−0.016 (3)	−0.001 (3)	−0.010 (4)
N6	0.037 (4)	0.049 (5)	0.079 (6)	−0.006 (4)	−0.005 (4)	0.001 (4)
C1	0.037 (4)	0.032 (5)	0.030 (4)	−0.016 (4)	0.013 (3)	−0.014 (3)
C2	0.039 (5)	0.027 (5)	0.051 (5)	−0.010 (4)	0.006 (4)	−0.014 (4)
C3	0.056 (6)	0.034 (5)	0.058 (6)	−0.024 (5)	0.026 (5)	−0.024 (4)
C4	0.085 (7)	0.038 (5)	0.024 (5)	−0.027 (5)	0.019 (4)	−0.012 (4)
C5	0.055 (5)	0.050 (6)	0.050 (6)	−0.025 (5)	−0.006 (4)	−0.027 (4)
C6	0.016 (4)	0.027 (4)	0.033 (4)	−0.007 (3)	0.000 (3)	0.008 (3)
C7	0.015 (3)	0.029 (4)	0.030 (4)	−0.009 (3)	0.008 (3)	−0.009 (3)
C8	0.015 (4)	0.041 (5)	0.048 (5)	−0.010 (4)	−0.005 (3)	0.021 (4)
C9	0.017 (4)	0.019 (4)	0.079 (7)	−0.007 (3)	0.001 (4)	0.002 (4)
C10	0.023 (4)	0.042 (5)	0.042 (5)	−0.019 (4)	0.005 (3)	−0.016 (4)
C11	0.059 (6)	0.049 (6)	0.068 (7)	−0.033 (5)	0.017 (5)	−0.015 (5)
C12	0.058 (6)	0.042 (6)	0.086 (8)	0.004 (5)	−0.002 (5)	−0.016 (5)
C13	0.054 (6)	0.052 (7)	0.18 (1)	−0.023 (6)	0.061 (7)	−0.060 (7)
C14	0.22 (1)	0.078 (8)	0.050 (7)	−0.065 (9)	0.066 (8)	−0.031 (6)
C15	0.13 (1)	0.067 (8)	0.096 (9)	−0.035 (7)	−0.046 (7)	−0.026 (6)
C16	0.019 (4)	0.059 (6)	0.052 (5)	−0.011 (4)	−0.005 (4)	0.014 (5)
C17	0.038 (5)	0.059 (6)	0.060 (6)	−0.024 (5)	0.025 (4)	−0.021 (5)
C18	0.045 (5)	0.134 (10)	0.056 (6)	−0.056 (6)	−0.013 (4)	0.050 (6)
C19	0.048 (6)	0.039 (6)	0.18 (1)	−0.030 (5)	0.005 (7)	0.004 (7)
C20	0.039 (5)	0.098 (8)	0.081 (7)	−0.050 (5)	0.020 (5)	−0.046 (6)
C21	0.022 (4)	0.031 (4)	0.034 (5)	−0.010 (3)	−0.002 (3)	−0.010 (4)
C22	0.046 (5)	0.033 (5)	0.021 (4)	−0.016 (4)	−0.005 (3)	−0.004 (3)
C23	0.052 (5)	0.035 (5)	0.023 (4)	−0.019 (4)	0.001 (4)	−0.004 (3)
C24	0.036 (4)	0.038 (5)	0.026 (4)	−0.020 (4)	0.009 (3)	−0.012 (3)
C25	0.024 (4)	0.030 (4)	0.025 (4)	−0.014 (3)	0.010 (3)	−0.006 (3)
C26	0.026 (4)	0.028 (4)	0.026 (4)	−0.015 (3)	0.004 (3)	−0.003 (3)
C27	0.025 (4)	0.036 (5)	0.034 (4)	−0.016 (4)	0.010 (3)	−0.003 (4)
C28	0.020 (4)	0.038 (5)	0.067 (6)	−0.016 (4)	0.002 (4)	−0.005 (4)
C29	0.027 (4)	0.043 (5)	0.052 (5)	−0.023 (4)	−0.008 (4)	0.006 (4)
C30	0.036 (4)	0.042 (5)	0.030 (4)	−0.024 (4)	−0.006 (3)	0.006 (4)
C31	0.059 (6)	0.044 (6)	0.043 (5)	−0.021 (5)	−0.005 (4)	0.001 (4)
C32	0.044 (5)	0.064 (6)	0.070 (7)	−0.034 (5)	−0.026 (5)	0.016 (5)
C33	0.019 (4)	0.026 (4)	0.034 (4)	−0.010 (3)	0.002 (3)	−0.001 (3)
C34	0.022 (4)	0.046 (5)	0.027 (4)	−0.015 (4)	0.001 (3)	−0.003 (4)
C35	0.032 (4)	0.046 (5)	0.021 (4)	−0.016 (4)	0.002 (3)	−0.006 (4)
C36	0.029 (4)	0.035 (5)	0.033 (4)	−0.012 (4)	0.004 (3)	−0.013 (4)
C37	0.021 (3)	0.026 (4)	0.032 (4)	−0.015 (3)	0.004 (3)	−0.005 (3)
C38	0.018 (4)	0.028 (4)	0.038 (5)	−0.010 (3)	−0.001 (3)	−0.005 (3)
C39	0.026 (4)	0.035 (5)	0.040 (5)	−0.008 (4)	0.004 (4)	−0.011 (4)
C40	0.031 (4)	0.024 (4)	0.049 (5)	0.002 (4)	0.006 (4)	−0.016 (4)
C41	0.024 (4)	0.025 (4)	0.053 (5)	−0.006 (3)	−0.008 (4)	−0.001 (4)
C42	0.021 (4)	0.028 (4)	0.030 (4)	−0.004 (3)	−0.002 (3)	−0.003 (3)
C43	0.043 (5)	0.046 (5)	0.026 (4)	−0.016 (4)	0.004 (4)	0.002 (4)
C44	0.043 (5)	0.040 (5)	0.057 (6)	0.003 (4)	−0.009 (4)	−0.006 (4)
C45	0.031 (4)	0.040 (5)	0.052 (6)	−0.019 (4)	−0.005 (4)	−0.009 (4)
C46	0.039 (5)	0.041 (5)	0.048 (5)	−0.019 (4)	0.003 (4)	−0.013 (4)
C47	0.056 (6)	0.062 (7)	0.059 (7)	−0.015 (5)	0.021 (5)	−0.022 (5)
C48	0.042 (5)	0.047 (6)	0.061 (7)	−0.007 (4)	0.008 (5)	−0.014 (5)
C49	0.028 (4)	0.033 (5)	0.054 (6)	−0.013 (4)	0.001 (4)	−0.006 (4)
C50	0.026 (4)	0.035 (5)	0.064 (6)	−0.008 (4)	0.006 (4)	−0.008 (4)
C51	0.039 (5)	0.051 (6)	0.067 (7)	−0.020 (5)	−0.001 (5)	0.008 (5)
C52	0.053 (6)	0.061 (7)	0.083 (8)	−0.026 (5)	−0.008 (5)	0.013 (6)
C53	0.066 (7)	0.046 (6)	0.068 (7)	−0.008 (5)	−0.011 (6)	0.014 (5)
C54	0.039 (5)	0.046 (6)	0.097 (9)	−0.001 (5)	−0.013 (6)	−0.009 (6)
C55	0.061 (6)	0.078 (7)	0.047 (6)	−0.038 (6)	0.001 (5)	−0.009 (5)
C56	0.104 (9)	0.058 (7)	0.092 (9)	−0.022 (7)	−0.027 (7)	0.016 (6)
C57	0.024 (4)	0.030 (4)	0.021 (4)	−0.005 (3)	−0.010 (3)	0.008 (3)
C58	0.027 (4)	0.033 (4)	0.023 (4)	−0.009 (3)	−0.004 (3)	0.000 (3)
C59	0.026 (4)	0.051 (6)	0.031 (5)	−0.008 (4)	−0.002 (3)	0.002 (4)
C60	0.036 (5)	0.061 (6)	0.040 (5)	−0.026 (5)	0.002 (4)	0.011 (4)
C61	0.044 (5)	0.043 (5)	0.049 (5)	−0.025 (4)	−0.004 (4)	0.015 (4)
C62	0.021 (4)	0.037 (5)	0.032 (4)	−0.002 (3)	−0.003 (3)	0.004 (4)
C63	0.022 (4)	0.028 (4)	0.020 (4)	−0.008 (3)	0.004 (3)	−0.004 (3)
C64	0.021 (4)	0.028 (4)	0.023 (4)	−0.005 (3)	0.002 (3)	0.000 (3)
C65	0.035 (4)	0.047 (5)	0.019 (4)	−0.024 (4)	0.001 (3)	−0.002 (4)
C66	0.041 (4)	0.025 (4)	0.035 (5)	−0.015 (4)	0.010 (4)	−0.010 (4)
C67	0.035 (4)	0.028 (5)	0.030 (4)	0.001 (4)	0.004 (3)	0.003 (4)
C68	0.027 (4)	0.030 (4)	0.020 (4)	−0.004 (3)	−0.006 (3)	0.002 (3)
C69	0.023 (4)	0.018 (4)	0.024 (4)	−0.004 (3)	0.002 (3)	0.001 (3)
C70	0.031 (4)	0.026 (4)	0.026 (4)	−0.008 (3)	−0.003 (3)	0.002 (3)
C71	0.022 (4)	0.038 (5)	0.041 (5)	−0.013 (4)	0.002 (3)	−0.005 (4)
C72	0.022 (4)	0.020 (4)	0.044 (5)	−0.005 (3)	−0.012 (3)	−0.001 (3)
C73	0.035 (4)	0.032 (5)	0.033 (4)	−0.006 (4)	−0.004 (3)	0.009 (4)
C74	0.022 (4)	0.025 (4)	0.030 (4)	−0.001 (3)	0.000 (3)	0.010 (3)
C75	0.017 (3)	0.020 (4)	0.028 (4)	−0.001 (3)	0.003 (3)	−0.004 (3)
C76	0.022 (4)	0.018 (4)	0.028 (4)	−0.001 (3)	−0.001 (3)	0.000 (3)
C77	0.027 (4)	0.029 (5)	0.030 (4)	0.004 (3)	0.005 (3)	0.000 (3)
C78	0.036 (4)	0.036 (5)	0.021 (4)	0.001 (4)	0.000 (3)	−0.004 (4)
C79	0.036 (5)	0.049 (5)	0.037 (5)	−0.016 (4)	−0.009 (4)	−0.006 (4)
C80	0.030 (4)	0.037 (5)	0.029 (4)	−0.009 (4)	−0.002 (3)	−0.003 (4)
C81	0.057 (6)	0.076 (7)	0.023 (5)	−0.037 (6)	−0.001 (4)	0.007 (5)
B1	0.023 (4)	0.022 (5)	0.020 (4)	−0.005 (4)	−0.001 (3)	−0.003 (3)

### Geometric parameters (Å, °)

**Table:**

Yb1—Yb2	3.5990 (4)	C61—C62	1.382 (10)
Yb1—O1	2.255 (4)	C63—C64	1.404 (9)
Yb1—O2	2.207 (4)	C63—C68	1.393 (9)
Yb1—O3	2.361 (4)	C63—B1	1.65 (1)
Yb1—N1	2.408 (5)	C64—C65	1.384 (10)
Yb1—N2	2.424 (5)	C65—C66	1.377 (10)
Yb1—C1	2.651 (7)	C66—C67	1.39 (1)
Yb1—C2	2.704 (8)	C67—C68	1.40 (1)
Yb1—C3	2.660 (8)	C69—C70	1.398 (9)
Yb1—C4	2.635 (8)	C69—C74	1.396 (9)
Yb1—C5	2.622 (8)	C69—B1	1.645 (10)
Yb1—Cg1	2.3750 (3)	C70—C71	1.386 (9)
Yb2—O1	2.295 (5)	C71—C72	1.389 (10)
Yb2—O2	2.214 (4)	C72—C73	1.370 (10)
Yb2—O4	2.332 (5)	C73—C74	1.392 (9)
Yb2—N3	2.408 (6)	C75—C76	1.397 (9)
Yb2—N4	2.389 (6)	C75—C80	1.393 (10)
Yb2—C6	2.611 (6)	C75—B1	1.656 (10)
Yb2—C7	2.598 (6)	C76—C77	1.389 (10)
Yb2—C8	2.648 (7)	C77—C78	1.367 (11)
Yb2—C9	2.698 (7)	C78—C79	1.37 (1)
Yb2—C10	2.677 (7)	C79—C80	1.398 (10)
Yb2—Cg2	2.3585 (3)	O1—H1	0.791
S1—O3	1.450 (5)	O2—H2	0.949
S1—O4	1.460 (5)	C11—H3	0.948
S1—O5	1.410 (5)	C11—H4	0.948
S1—C81	1.821 (8)	C11—H5	0.949
F1—C81	1.324 (9)	C12—H6	0.947
F2—C81	1.341 (10)	C12—H7	0.951
F3—C81	1.319 (10)	C12—H8	0.948
N1—C21	1.347 (8)	C13—H9	0.949
N1—C25	1.342 (8)	C13—H10	0.950
N2—C26	1.349 (8)	C13—H11	0.950
N2—C30	1.351 (9)	C14—H12	0.954
N3—C33	1.333 (8)	C14—H13	0.942
N3—C37	1.361 (8)	C14—H14	0.951
N4—C38	1.346 (9)	C15—H15	0.948
N4—C42	1.347 (8)	C15—H16	0.947
N5—C45	1.327 (10)	C15—H17	0.951
N5—C49	1.355 (9)	C16—H18	0.949
N6—C50	1.353 (9)	C16—H19	0.947
N6—C54	1.34 (1)	C16—H20	0.951
C1—C2	1.43 (1)	C17—H21	0.950
C1—C5	1.39 (1)	C17—H22	0.950
C1—C11	1.51 (1)	C17—H23	0.950
C1—Cg1	1.188 (7)	C18—H24	0.951
C2—C3	1.38 (1)	C18—H25	0.947
C2—C12	1.49 (1)	C18—H26	0.949
C2—Cg1	1.200 (9)	C19—H27	0.951
C3—C4	1.39 (1)	C19—H28	0.952
C3—C13	1.54 (1)	C19—H29	0.947
C3—Cg1	1.181 (8)	C20—H30	0.949
C4—C5	1.44 (1)	C20—H31	0.951
C4—C14	1.51 (1)	C20—H32	0.949
C4—Cg1	1.197 (8)	C21—H33	0.951
C5—C15	1.51 (1)	C23—H34	0.951
C5—Cg1	1.207 (8)	C24—H35	0.951
C6—C7	1.424 (9)	C27—H36	0.951
C6—C10	1.401 (10)	C28—H37	0.950
C6—C16	1.494 (10)	C30—H38	0.953
C6—Cg2	1.195 (7)	C31—H39	0.949
C7—C8	1.406 (10)	C31—H40	0.949
C7—C17	1.51 (1)	C31—H41	0.950
C7—Cg2	1.210 (7)	C32—H42	0.948
C8—C9	1.41 (1)	C32—H43	0.948
C8—C18	1.53 (1)	C32—H44	0.951
C8—Cg2	1.201 (8)	C33—H45	0.951
C9—C10	1.41 (1)	C35—H46	0.950
C9—C19	1.49 (1)	C36—H47	0.951
C9—Cg2	1.204 (8)	C39—H48	0.950
C10—C20	1.49 (1)	C40—H49	0.950
C10—Cg2	1.189 (8)	C42—H50	0.953
C21—C22	1.401 (9)	C43—H51	0.949
C22—C23	1.38 (1)	C43—H52	0.950
C22—C31	1.50 (1)	C43—H53	0.949
C23—C24	1.367 (10)	C44—H54	0.950
C24—C25	1.394 (9)	C44—H55	0.947
C25—C26	1.474 (9)	C44—H56	0.949
C26—C27	1.403 (9)	C45—H57	0.951
C27—C28	1.37 (1)	C47—H58	0.950
C28—C29	1.37 (1)	C48—H59	0.950
C29—C30	1.389 (10)	C51—H60	0.952
C29—C32	1.51 (1)	C52—H61	0.953
C33—C34	1.368 (10)	C54—H62	0.951
C34—C35	1.40 (1)	C55—H63	0.950
C34—C43	1.51 (1)	C55—H64	0.948
C35—C36	1.381 (10)	C55—H65	0.950
C36—C37	1.394 (9)	C56—H66	0.947
C37—C38	1.496 (9)	C56—H67	0.947
C38—C39	1.384 (10)	C56—H68	0.949
C39—C40	1.37 (1)	C58—H69	0.951
C40—C41	1.38 (1)	C59—H70	0.947
C41—C42	1.378 (10)	C60—H71	0.950
C41—C44	1.52 (1)	C61—H72	0.951
C45—C46	1.40 (1)	C62—H73	0.951
C46—C47	1.37 (1)	C64—H74	0.951
C46—C55	1.48 (1)	C65—H75	0.952
C47—C48	1.39 (1)	C66—H76	0.950
C48—C49	1.39 (1)	C67—H77	0.952
C49—C50	1.46 (1)	C68—H78	0.951
C50—C51	1.40 (1)	C70—H79	0.952
C51—C52	1.40 (1)	C71—H80	0.952
C52—C53	1.39 (1)	C72—H81	0.950
C53—C54	1.37 (1)	C73—H82	0.951
C53—C56	1.50 (1)	C74—H83	0.950
C57—C58	1.407 (9)	C76—H84	0.951
C57—C62	1.398 (10)	C77—H85	0.951
C57—B1	1.652 (10)	C78—H86	0.951
C58—C59	1.382 (10)	C79—H87	0.950
C59—C60	1.38 (1)	C80—H88	0.950
C60—C61	1.37 (1)		
			
Yb1···Yb2	3.5990 (4)	C16···C77^iv^	3.54 (1)
Yb1···C1	2.651 (7)	C18···C56^iii^	3.57 (1)
Yb1···C2	2.704 (8)	C23···C70	3.49 (1)
Yb1···C3	2.660 (8)	C23···C71	3.56 (1)
Yb1···C4	2.635 (8)	C23···C64	3.58 (1)
Yb1···C5	2.622 (8)	C24···C64	3.501 (9)
Yb2···C6	2.611 (6)	C25···C45	3.36 (1)
Yb2···C7	2.598 (6)	C26···C45	3.34 (1)
Yb2···C8	2.648 (7)	C27···C45	3.47 (1)
Yb2···C9	2.698 (7)	C27···C49	3.57 (1)
Yb2···C10	2.677 (7)	C28···C49	3.46 (1)
F1···C15^i^	3.43 (1)	C28···C50	3.58 (1)
F2···C17^ii^	3.287 (9)	C29···C51	3.48 (1)
F3···C56^iii^	3.57 (1)	C29···C50	3.52 (1)
O2···N5	3.012 (8)	C32···C52	3.47 (1)
O2···C45	3.406 (10)	C32···C51	3.53 (1)
O5···C52	3.18 (1)	C32···C53	3.60 (1)
O5···C51	3.25 (1)	C36···C45	3.60 (1)
N2···N5	3.540 (9)	C37···C45	3.258 (9)
N3···C45	3.529 (9)	C38···C45	3.470 (10)
N5···C26	3.291 (9)	C39···C70^v^	3.34 (1)
N5···C38	3.314 (9)	C39···C71^v^	3.52 (1)
N5···C27	3.325 (10)	C41···C51	3.48 (1)
N5···C39	3.52 (1)	C42···C51	3.58 (1)
C6···C28^iv^	3.491 (9)	C43···C58^vi^	3.59 (1)
C7···C32^iv^	3.568 (10)	C44···C78^v^	3.56 (1)
C16···C28^iv^	3.49 (1)		
			
Yb2—Yb1—O1	38.1 (1)	N3—C37—C36	120.2 (6)
Yb2—Yb1—O2	35.6 (1)	N3—C37—C38	115.7 (6)
Yb2—Yb1—O3	75.5 (1)	C36—C37—C38	124.1 (7)
Yb2—Yb1—N1	97.5 (1)	N4—C38—C37	115.3 (6)
Yb2—Yb1—N2	112.9 (1)	N4—C38—C39	122.0 (7)
Yb2—Yb1—C1	157.9 (2)	C37—C38—C39	122.7 (7)
Yb2—Yb1—C2	127.4 (2)	C38—C39—C40	118.9 (7)
Yb2—Yb1—C3	113.8 (2)	C39—C40—C41	120.8 (7)
Yb2—Yb1—C4	125.3 (2)	C40—C41—C42	116.4 (7)
Yb2—Yb1—C5	156.2 (2)	C40—C41—C44	123.2 (7)
Yb2—Yb1—Cg1	140.09 (1)	C42—C41—C44	120.4 (8)
O1—Yb1—O2	71.5 (2)	N4—C42—C41	124.5 (7)
O1—Yb1—O3	95.5 (2)	N5—C45—C46	125.7 (8)
O1—Yb1—N1	93.7 (2)	C45—C46—C47	115.7 (8)
O1—Yb1—N2	145.1 (2)	C45—C46—C55	120.8 (8)
O1—Yb1—C1	120.0 (2)	C47—C46—C55	123.4 (8)
O1—Yb1—C2	89.2 (2)	C46—C47—C48	120.7 (8)
O1—Yb1—C3	80.0 (2)	C47—C48—C49	119.0 (8)
O1—Yb1—C4	102.7 (2)	N5—C49—C48	121.9 (8)
O1—Yb1—C5	131.0 (2)	N5—C49—C50	116.7 (7)
O1—Yb1—Cg1	105.3 (1)	C48—C49—C50	121.4 (8)
O2—Yb1—O3	76.4 (2)	N6—C50—C49	116.6 (8)
O2—Yb1—N1	83.4 (2)	N6—C50—C51	120.8 (8)
O2—Yb1—N2	77.3 (2)	C49—C50—C51	122.6 (7)
O2—Yb1—C1	158.7 (2)	C50—C51—C52	119.9 (8)
O2—Yb1—C2	154.8 (2)	C51—C52—C53	119.2 (9)
O2—Yb1—C3	149.2 (2)	C52—C53—C54	116.2 (9)
O2—Yb1—C4	148.5 (2)	C52—C53—C56	121 (1)
O2—Yb1—C5	153.9 (2)	C54—C53—C56	121.9 (10)
O2—Yb1—Cg1	174.7 (1)	N6—C54—C53	126.7 (9)
O3—Yb1—N1	153.8 (2)	C58—C57—C62	114.6 (6)
O3—Yb1—N2	92.0 (2)	C58—C57—B1	122.7 (6)
O3—Yb1—C1	117.4 (2)	C62—C57—B1	122.6 (6)
O3—Yb1—C2	122.9 (2)	C57—C58—C59	122.8 (7)
O3—Yb1—C3	95.4 (2)	C58—C59—C60	120.3 (7)
O3—Yb1—C4	73.4 (2)	C59—C60—C61	118.7 (7)
O3—Yb1—C5	87.0 (2)	C60—C61—C62	120.9 (8)
O3—Yb1—Cg1	99.9 (1)	C57—C62—C61	122.8 (7)
N1—Yb1—N2	67.0 (2)	C64—C63—C68	114.5 (6)
N1—Yb1—C1	78.2 (2)	C64—C63—B1	123.0 (6)
N1—Yb1—C2	81.7 (2)	C68—C63—B1	122.5 (6)
N1—Yb1—C3	110.4 (2)	C63—C64—C65	123.4 (6)
N1—Yb1—C4	128.1 (2)	C64—C65—C66	120.1 (7)
N1—Yb1—C5	105.1 (2)	C65—C66—C67	119.1 (7)
N1—Yb1—Cg1	101.2 (1)	C66—C67—C68	119.5 (7)
N2—Yb1—C1	85.7 (2)	C63—C68—C67	123.4 (7)
N2—Yb1—C2	114.8 (2)	C70—C69—C74	114.7 (6)
N2—Yb1—C3	133.2 (2)	C70—C69—B1	122.4 (6)
N2—Yb1—C4	112.1 (3)	C74—C69—B1	122.5 (6)
N2—Yb1—C5	83.4 (2)	C69—C70—C71	123.1 (6)
N2—Yb1—Cg1	106.9 (1)	C70—C71—C72	120.2 (6)
C1—Yb1—C2	30.9 (2)	C71—C72—C73	118.5 (6)
C1—Yb1—C3	50.2 (2)	C72—C73—C74	120.5 (6)
C1—Yb1—C4	50.9 (2)	C69—C74—C73	123.0 (6)
C1—Yb1—C5	30.5 (2)	C76—C75—C80	114.6 (6)
C1—Yb1—Cg1	26.6 (1)	C76—C75—B1	122.6 (6)
C2—Yb1—C3	29.8 (2)	C80—C75—B1	122.8 (6)
C2—Yb1—C4	50.3 (3)	C75—C76—C77	123.6 (7)
C2—Yb1—C5	50.9 (3)	C76—C77—C78	119.7 (7)
C2—Yb1—Cg1	26.3 (2)	C77—C78—C79	119.0 (7)
C3—Yb1—C4	30.4 (3)	C78—C79—C80	120.7 (7)
C3—Yb1—C5	51.1 (3)	C75—C80—C79	122.5 (7)
C3—Yb1—Cg1	26.4 (2)	S1—C81—F1	111.4 (6)
C4—Yb1—C5	31.8 (3)	S1—C81—F2	110.7 (5)
C4—Yb1—Cg1	27.0 (2)	S1—C81—F3	111.1 (7)
C5—Yb1—Cg1	27.4 (2)	F1—C81—F2	107.5 (8)
Yb1—Yb2—O1	37.3 (1)	F1—C81—F3	108.1 (7)
Yb1—Yb2—O2	35.5 (1)	F2—C81—F3	107.8 (7)
Yb1—Yb2—O4	75.7 (1)	Yb1—Cg1—C1	89.8 (3)
Yb1—Yb2—N3	98.2 (1)	Yb1—Cg1—C2	92.3 (4)
Yb1—Yb2—N4	116.7 (1)	Yb1—Cg1—C3	90.4 (4)
Yb1—Yb2—C6	121.5 (2)	Yb1—Cg1—C4	88.7 (4)
Yb1—Yb2—C7	113.0 (2)	Yb1—Cg1—C5	87.8 (4)
Yb1—Yb2—C8	131.9 (2)	C1—Cg1—C2	73.3 (5)
Yb1—Yb2—C9	162.4 (2)	C1—Cg1—C3	144.1 (6)
Yb1—Yb2—C10	150.0 (2)	C1—Cg1—C4	144.4 (6)
Yb1—Yb2—Cg2	140.08 (1)	C1—Cg1—C5	70.8 (5)
O1—Yb2—O2	70.7 (2)	C2—Cg1—C3	70.9 (6)
O1—Yb2—O4	94.6 (2)	C2—Cg1—C4	142.3 (6)
O1—Yb2—N3	97.0 (2)	C2—Cg1—C5	144.1 (6)
O1—Yb2—N4	150.1 (2)	C3—Cg1—C4	71.5 (6)
O1—Yb2—C6	84.2 (2)	C3—Cg1—C5	145.1 (6)
O1—Yb2—C7	81.8 (2)	C4—Cg1—C5	73.6 (6)
O1—Yb2—C8	110.0 (2)	Yb2—Cg2—C6	88.2 (3)
O1—Yb2—C9	131.8 (2)	Yb2—Cg2—C7	87.2 (3)
O1—Yb2—C10	113.4 (2)	Yb2—Cg2—C8	90.1 (3)
O1—Yb2—Cg2	105.4 (1)	Yb2—Cg2—C9	92.7 (3)
O2—Yb2—O4	77.1 (1)	Yb2—Cg2—C10	91.9 (3)
O2—Yb2—N3	81.8 (2)	C6—Cg2—C7	72.6 (5)
O2—Yb2—N4	81.5 (2)	C6—Cg2—C8	143.9 (5)
O2—Yb2—C6	151.0 (2)	C6—Cg2—C9	144.3 (6)
O2—Yb2—C7	147.7 (2)	C6—Cg2—C10	72.0 (5)
O2—Yb2—C8	151.4 (2)	C7—Cg2—C8	71.3 (5)
O2—Yb2—C9	157.2 (2)	C7—Cg2—C9	143.1 (6)
O2—Yb2—C10	157.1 (2)	C7—Cg2—C10	144.6 (5)
O2—Yb2—Cg2	175.4 (1)	C8—Cg2—C9	71.8 (5)
O4—Yb2—N3	151.0 (2)	C8—Cg2—C10	144.1 (6)
O4—Yb2—N4	89.3 (2)	C9—Cg2—C10	72.3 (5)
O4—Yb2—C6	120.3 (2)	C57—B1—C63	108.5 (5)
O4—Yb2—C7	88.9 (2)	C57—B1—C69	111.9 (5)
O4—Yb2—C8	74.3 (2)	C57—B1—C75	108.7 (6)
O4—Yb2—C9	94.6 (2)	C63—B1—C69	107.0 (5)
O4—Yb2—C10	123.4 (2)	C63—B1—C75	109.7 (5)
O4—Yb2—Cg2	101.1 (1)	C69—B1—C75	110.9 (5)
N3—Yb2—N4	67.8 (2)	Yb1—O1—H1	121.540
N3—Yb2—C6	87.3 (2)	Yb2—O1—H1	128.794
N3—Yb2—C7	119.1 (2)	Yb1—O2—H2	124.632
N3—Yb2—C8	125.3 (2)	Yb2—O2—H2	122.142
N3—Yb2—C9	97.3 (2)	C1—C11—H3	109.167
N3—Yb2—C10	75.4 (2)	C1—C11—H4	109.215
N3—Yb2—Cg2	101.3 (1)	C1—C11—H5	109.165
N4—Yb2—C6	119.0 (2)	H3—C11—H4	109.783
N4—Yb2—C7	128.0 (2)	H3—C11—H5	109.734
N4—Yb2—C8	99.6 (2)	H4—C11—H5	109.759
N4—Yb2—C9	77.2 (2)	C2—C12—H6	109.364
N4—Yb2—C10	88.4 (2)	C2—C12—H7	109.062
N4—Yb2—Cg2	102.9 (1)	C2—C12—H8	109.305
C6—Yb2—C7	31.7 (2)	H6—C12—H7	109.628
C6—Yb2—C8	51.3 (2)	H6—C12—H8	109.887
C6—Yb2—C9	50.9 (2)	H7—C12—H8	109.578
C6—Yb2—C10	30.7 (2)	C3—C13—H9	109.411
C6—Yb2—Cg2	27.2 (1)	C3—C13—H10	109.362
C7—Yb2—C8	31.1 (2)	C3—C13—H11	109.458
C7—Yb2—C9	51.2 (2)	H9—C13—H10	109.590
C7—Yb2—C10	51.3 (2)	H9—C13—H11	109.557
C7—Yb2—Cg2	27.7 (2)	H10—C13—H11	109.448
C8—Yb2—C9	30.6 (2)	C4—C14—H12	110.000
C8—Yb2—C10	50.6 (2)	C4—C14—H13	109.703
C8—Yb2—Cg2	27.0 (2)	C4—C14—H14	109.135
C9—Yb2—C10	30.5 (2)	H12—C14—H13	109.867
C9—Yb2—Cg2	26.5 (2)	H12—C14—H14	109.049
C10—Yb2—Cg2	26.4 (2)	H13—C14—H14	109.000
O3—S1—O4	113.4 (3)	C5—C15—H15	109.284
O3—S1—O5	115.4 (3)	C5—C15—H16	109.316
O3—S1—C81	103.5 (4)	C5—C15—H17	109.089
O4—S1—O5	115.5 (3)	H15—C15—H16	109.915
O4—S1—C81	102.1 (3)	H15—C15—H17	109.546
O5—S1—C81	104.6 (4)	H16—C15—H17	109.672
Yb1—O1—Yb2	104.5 (2)	C6—C16—H18	109.371
Yb1—O2—Yb2	109.0 (2)	C6—C16—H19	109.379
Yb1—O3—S1	132.4 (3)	C6—C16—H20	109.227
Yb2—O4—S1	132.6 (3)	H18—C16—H19	109.773
Yb1—N1—C21	121.5 (4)	H18—C16—H20	109.474
Yb1—N1—C25	120.3 (4)	H19—C16—H20	109.601
C21—N1—C25	117.5 (6)	C7—C17—H21	109.431
Yb1—N2—C26	119.4 (4)	C7—C17—H22	109.458
Yb1—N2—C30	121.0 (4)	C7—C17—H23	109.427
C26—N2—C30	118.5 (6)	H21—C17—H22	109.507
Yb2—N3—C33	122.0 (5)	H21—C17—H23	109.479
Yb2—N3—C37	119.1 (4)	H22—C17—H23	109.524
C33—N3—C37	118.3 (6)	C8—C18—H24	109.203
Yb2—N4—C38	120.9 (5)	C8—C18—H25	109.365
Yb2—N4—C42	121.2 (5)	C8—C18—H26	109.271
C38—N4—C42	117.5 (6)	H24—C18—H25	109.666
C45—N5—C49	116.9 (7)	H24—C18—H26	109.522
C50—N6—C54	117.1 (8)	H25—C18—H26	109.798
Yb1—C1—C2	76.6 (4)	C9—C19—H27	109.391
Yb1—C1—C5	73.6 (4)	C9—C19—H28	109.384
Yb1—C1—C11	122.9 (5)	C9—C19—H29	109.613
Yb1—C1—Cg1	63.6 (3)	H27—C19—H28	109.241
C2—C1—C5	109.0 (7)	H27—C19—H29	109.664
C2—C1—C11	123.5 (7)	H28—C19—H29	109.534
C2—C1—Cg1	53.7 (4)	C10—C20—H30	109.492
C5—C1—C11	126.9 (8)	C10—C20—H31	109.366
C5—C1—Cg1	55.3 (5)	C10—C20—H32	109.468
C11—C1—Cg1	173.1 (7)	H30—C20—H31	109.459
Yb1—C2—C1	72.5 (4)	H30—C20—H32	109.606
Yb1—C2—C3	73.3 (5)	H31—C20—H32	109.437
Yb1—C2—C12	125.6 (6)	N1—C21—H33	117.703
Yb1—C2—Cg1	61.4 (3)	C22—C21—H33	117.715
C1—C2—C3	106.9 (7)	C22—C23—H34	119.482
C1—C2—C12	126.8 (8)	C24—C23—H34	119.511
C1—C2—Cg1	52.9 (4)	C23—C24—H35	120.214
C3—C2—C12	125.9 (8)	C25—C24—H35	120.294
C3—C2—Cg1	53.9 (5)	C26—C27—H36	120.325
C12—C2—Cg1	173.0 (8)	C28—C27—H36	120.143
Yb1—C3—C2	76.9 (5)	C27—C28—H37	119.492
Yb1—C3—C4	73.8 (5)	C29—C28—H37	119.318
Yb1—C3—C13	121.2 (5)	N2—C30—H38	118.164
Yb1—C3—Cg1	63.2 (3)	C29—C30—H38	117.829
C2—C3—C4	110.0 (7)	C22—C31—H39	109.386
C2—C3—C13	124.6 (9)	C22—C31—H40	109.420
C2—C3—Cg1	55.2 (5)	C22—C31—H41	109.335
C4—C3—C13	125.0 (9)	H39—C31—H40	109.645
C4—C3—Cg1	54.8 (5)	H39—C31—H41	109.525
C13—C3—Cg1	175.6 (8)	H40—C31—H41	109.515
Yb1—C4—C3	75.8 (5)	C29—C32—H42	109.376
Yb1—C4—C5	73.6 (4)	C29—C32—H43	109.447
Yb1—C4—C14	122.6 (6)	C29—C32—H44	109.149
Yb1—C4—Cg1	64.3 (3)	H42—C32—H43	109.803
C3—C4—C5	107.2 (7)	H42—C32—H44	109.524
C3—C4—C14	126.6 (10)	H43—C32—H44	109.525
C3—C4—Cg1	53.7 (5)	N3—C33—H45	117.409
C5—C4—C14	126.2 (9)	C34—C33—H45	117.229
C5—C4—Cg1	53.5 (5)	C34—C35—H46	120.014
C14—C4—Cg1	173.0 (8)	C36—C35—H46	120.237
Yb1—C5—C1	75.9 (4)	C35—C36—H47	119.943
Yb1—C5—C4	74.6 (5)	C37—C36—H47	120.169
Yb1—C5—C15	120.4 (6)	C38—C39—H48	120.540
Yb1—C5—Cg1	64.9 (3)	C40—C39—H48	120.576
C1—C5—C4	106.9 (7)	C39—C40—H49	119.541
C1—C5—C15	126.9 (9)	C41—C40—H49	119.676
C1—C5—Cg1	54.0 (5)	N4—C42—H50	117.842
C4—C5—C15	126.0 (9)	C41—C42—H50	117.695
C4—C5—Cg1	52.9 (5)	C34—C43—H51	109.468
C15—C5—Cg1	174.8 (8)	C34—C43—H52	109.352
Yb2—C6—C7	73.6 (4)	C34—C43—H53	109.424
Yb2—C6—C10	77.2 (4)	H51—C43—H52	109.498
Yb2—C6—C16	117.5 (4)	H51—C43—H53	109.592
Yb2—C6—Cg2	64.5 (3)	H52—C43—H53	109.493
C7—C6—C10	108.0 (6)	C41—C44—H54	109.206
C7—C6—C16	126.4 (7)	C41—C44—H55	109.348
C7—C6—Cg2	54.2 (4)	C41—C44—H56	109.230
C10—C6—C16	125.5 (7)	H54—C44—H55	109.699
C10—C6—Cg2	53.8 (4)	H54—C44—H56	109.574
C16—C6—Cg2	177.9 (6)	H55—C44—H56	109.767
Yb2—C7—C6	74.6 (4)	N5—C45—H57	117.152
Yb2—C7—C8	76.4 (4)	C46—C45—H57	117.165
Yb2—C7—C17	117.8 (4)	C46—C47—H58	119.674
Yb2—C7—Cg2	65.1 (3)	C48—C47—H58	119.577
C6—C7—C8	107.2 (6)	C47—C48—H59	120.534
C6—C7—C17	125.0 (7)	C49—C48—H59	120.433
C6—C7—Cg2	53.2 (4)	C50—C51—H60	120.009
C8—C7—C17	127.6 (7)	C52—C51—H60	120.063
C8—C7—Cg2	54.0 (4)	C51—C52—H61	120.157
C17—C7—Cg2	176.6 (6)	C53—C52—H61	120.656
Yb2—C8—C7	72.5 (4)	N6—C54—H62	116.848
Yb2—C8—C9	76.7 (4)	C53—C54—H62	116.441
Yb2—C8—C18	121.9 (5)	C46—C55—H63	109.214
Yb2—C8—Cg2	63.0 (3)	C46—C55—H64	109.455
C7—C8—C9	108.9 (7)	C46—C55—H65	109.231
C7—C8—C18	124.1 (8)	H63—C55—H64	109.712
C7—C8—Cg2	54.6 (4)	H63—C55—H65	109.518
C9—C8—C18	126.7 (8)	H64—C55—H65	109.695
C9—C8—Cg2	54.2 (5)	C53—C56—H66	109.093
C18—C8—Cg2	174.9 (6)	C53—C56—H67	109.227
Yb2—C9—C8	72.8 (4)	C53—C56—H68	109.036
Yb2—C9—C10	73.9 (4)	H66—C56—H67	109.917
Yb2—C9—C19	125.5 (5)	H66—C56—H68	109.781
Yb2—C9—Cg2	60.8 (3)	H67—C56—H68	109.766
C8—C9—C10	107.4 (7)	C57—C58—H69	118.639
C8—C9—C19	126.9 (9)	C59—C58—H69	118.541
C8—C9—Cg2	54.0 (4)	C58—C59—H70	120.060
C10—C9—C19	125.2 (9)	C60—C59—H70	119.669
C10—C9—Cg2	53.4 (4)	C59—C60—H71	120.611
C19—C9—Cg2	173.5 (7)	C61—C60—H71	120.712
Yb2—C10—C6	72.1 (4)	C60—C61—H72	119.646
Yb2—C10—C9	75.6 (4)	C62—C61—H72	119.500
Yb2—C10—C20	125.2 (5)	C57—C62—H73	118.530
Yb2—C10—Cg2	61.7 (3)	C61—C62—H73	118.653
C6—C10—C9	108.5 (7)	C63—C64—H74	118.309
C6—C10—C20	127.4 (8)	C65—C64—H74	118.285
C6—C10—Cg2	54.2 (4)	C64—C65—H75	119.870
C9—C10—C20	123.6 (8)	C66—C65—H75	120.014
C9—C10—Cg2	54.3 (5)	C65—C66—H76	120.419
C20—C10—Cg2	172.9 (7)	C67—C66—H76	120.507
N1—C21—C22	124.6 (6)	C66—C67—H77	120.262
C21—C22—C23	115.8 (7)	C68—C67—H77	120.238
C21—C22—C31	120.3 (7)	C63—C68—H78	118.264
C23—C22—C31	124.0 (7)	C67—C68—H78	118.335
C22—C23—C24	121.0 (7)	C69—C70—H79	118.484
C23—C24—C25	119.5 (6)	C71—C70—H79	118.434
N1—C25—C24	121.5 (6)	C70—C71—H80	119.908
N1—C25—C26	115.8 (6)	C72—C71—H80	119.917
C24—C25—C26	122.7 (6)	C71—C72—H81	120.599
N2—C26—C25	116.2 (6)	C73—C72—H81	120.888
N2—C26—C27	120.4 (6)	C72—C73—H82	119.666
C25—C26—C27	123.4 (6)	C74—C73—H82	119.810
C26—C27—C28	119.5 (7)	C69—C74—H83	118.579
C27—C28—C29	121.2 (7)	C73—C74—H83	118.435
C28—C29—C30	116.4 (7)	C75—C76—H84	118.169
C28—C29—C32	122.9 (7)	C77—C76—H84	118.232
C30—C29—C32	120.6 (7)	C76—C77—H85	120.041
N2—C30—C29	124.0 (7)	C78—C77—H85	120.279
N3—C33—C34	125.4 (7)	C77—C78—H86	120.272
C33—C34—C35	116.4 (7)	C79—C78—H86	120.772
C33—C34—C43	120.6 (7)	C78—C79—H87	119.632
C35—C34—C43	123.0 (7)	C80—C79—H87	119.680
C34—C35—C36	119.7 (7)	C75—C80—H88	118.663
C35—C36—C37	119.9 (7)	C79—C80—H88	118.869
			
N1—C25—C26—N2	13.1 (9)	N5—C49—C50—N6	169.5 (7)
N3—C37—C38—N4	−12.1 (8)		

Symmetry codes: (i) −*x*+1, −*y*+1, −*z*+1; (ii) −*x*+2, −*y*+1, −*z*+1; (iii) −*x*+1, −*y*+2, −*z*+1; (iv) *x*+1, *y*, *z*; (v) *x*, *y*+1, *z*; (vi) −*x*+1, −*y*+1, −*z*+2.
